# Segmental HOG: new descriptor for glomerulus detection in kidney microscopy image

**DOI:** 10.1186/s12859-015-0739-1

**Published:** 2015-09-30

**Authors:** Tsuyoshi Kato, Raissa Relator, Hayliang Ngouv, Yoshihiro Hirohashi, Osamu Takaki, Tetsuhiro Kakimoto, Kinya Okada

**Affiliations:** 1 Faculty of Science and Engineering, Gunma University, Kiryu-shi, Gunma, 376–8515 Japan; 2 Center for Informational Biology, Ochanomizu University, Bunkyo-ku, Tokyo, 112-8610 Japan; 3 Gunma University Hospital, Maebashi-shi, Gunma, 371–8511 Japan; 4 Research Division, Mitsubishi Tanabe Pharma Corporation, Toda-shi, Saitama, 335–8505 Japan

**Keywords:** Microscopy image analysis, Glomerulus detection, Computer vision, Support vector machine, Dynamic programming, Glomerular injury marker, Desmin immunostaining

## Abstract

**Background:**

The detection of the glomeruli is a key step in the histopathological evaluation of microscopic images of the kidneys. However, the task of automatic detection of the glomeruli poses challenges owing to the differences in their sizes and shapes in renal sections as well as the extensive variations in their intensities due to heterogeneity in immunohistochemistry staining.

Although the rectangular histogram of oriented gradients (Rectangular HOG) is a widely recognized powerful descriptor for general object detection, it shows many false positives owing to the aforementioned difficulties in the context of glomeruli detection.

**Results:**

A new descriptor referred to as Segmental HOG was developed to perform a comprehensive detection of hundreds of glomeruli in images of whole kidney sections. The new descriptor possesses flexible blocks that can be adaptively fitted to input images in order to acquire robustness for the detection of the glomeruli. Moreover, the novel segmentation technique employed herewith generates high-quality segmentation outputs, and the algorithm is assured to converge to an optimal solution. Consequently, experiments using real-world image data revealed that Segmental HOG achieved significant improvements in detection performance compared to Rectangular HOG.

**Conclusion:**

The proposed descriptor for glomeruli detection presents promising results, and it is expected to be useful in pathological evaluation.

## Background

The glomeruli in the kidneys act as a filtration barrier that retains higher molecular weight proteins in blood circulation. In various renal diseases, the glomerular filtration barrier can be damaged, resulting in protein leakage into urine, known as proteinuria. Therefore, the pathological changes in renal glomeruli of animal disease models can provide important information in screening compounds that target such diseases.

Our goal was to perform high-throughput detection of the glomeruli in highly enlarged microscopy images of animal disease models, whose sizes run up to the order of 10^8^ pixels. Although there are existing studies about the automatic analysis of the glomeruli in microscopic images of the kidneys [1, 2], the target images in these studies were obtained from human biopsy samples with relatively small sizes; therefore, they are not suitable for our purpose.

Compared to general object detection tasks, there are two particular obstacles in the case of glomeruli detection. The first obstacle arises from the non-rigid sizes and shapes of the targets in the images. Indeed, the glomeruli have a fixed size *in vivo*, although they swell to some extent in unfavorable situations such as hypertension [3] and diabetes [4]. In addition, the sizes of the glomeruli in a whole-kidney-section image could vary depending on where the cross-section was taken. The shape of the glomeruli is mostly spherical, making the boundaries circular. To obtain the boundaries, one might try to fit an ellipse to each glomerulus. However, this approach yields large estimation errors because each glomerulus is deformed to some extent.

The second difficulty arising in the glomerulus detection task is the high variation in staining intensity. On histological evaluation, immunohistochemistry is usually used to demonstrate the distribution and location of proteins in tissue sections. In our target images, sections were immunostained for desmin, a known glomerular injury marker. Therefore, some glomeruli are stained and some are not. As many glomeruli are partly stained, resulting in heterogeneously stained glomeruli, detection is more complicated. Furthermore, the stained tissues in the kidneys are not only from the glomeruli but also from other tissues such as the blood vessels.

To check the existence of a glomerulus at each location in a whole-kidney-section image, the sliding window technique is employed [5–8]. Using this procedure, a frame goes over the input image to check for the target object at every possible location; then, a descriptor of the sub-image is extracted.

Rectangular HOG (R-HOG) [9], a widely used and recognized efficient descriptor for object detection in the field of computer vision, is a potentially suitable candidate descriptor for glomeruli. It has the capacity to capture information about the magnitude of the gradients in the image. Therefore, this descriptor is robust to the change in intensities caused by the heterogeneity of the stained levels. Glomeruli are known to be composed of tightly packed cells, resulting in high gradients on images. Thus, a natural approach would be to use the magnitude of these gradients as features of the glomeruli. However, although we have previously attempted to directly exploit this attribute, we found the detection performance to be poor, resulting in many false positives and low recall. In addition to the magnitude of the gradients, their directions are also important to distinguish the glomeruli from the other tissues. Using R-HOG descriptors obtained from both the magnitude and the direction of the gradients, glomeruli detection performance results in recall values high enough to be useful for pathological evaluation. However, it appeared that R-HOG still has a considerable amount of false positives [10–12].

The high number of false positives in previous studies [10–12] can be ascribed to the condition that the standard HOG such as the R-HOG has a rigid block division. Owing to this rigidity, there are instances when a block is inside the glomerular area in a case and outside in another. Thus, extracted features from each block contain large differences, and robustness for the deformations of glomeruli is lost. Although there are several other known local descriptors such as scale-invariant feature transform (SIFT) image features [13], Haar-like features [8], and local binary patterns (LBP) [14], these do not possess a solution to be robust for deformed glomeruli for similar reasons.

In this study, we introduce flexible block division to the HOG descriptor to improve the detection performance and to reduce the number of false positives. A new feature, which we refer to as the Segmental HOG (S-HOG) descriptor, has been proposed for glomerulus detection. The block division of S-HOG is based on the estimated boundary of the glomerulus that is obtained via a segmentation algorithm, which has also been developed in this study. This renders the division of blocks to be more adaptable than the rigid block division of R-HOG, and allows feature vectors to clearly differentiate between the inside and the outside of the glomerulus. Moreover, because blocks are always within the glomerular area, gradient information in the same block between two glomeruli is expected to be more similar. Experiments revealed that the number of false positives was halved, keeping almost all true positives when using S-HOG compared to the R-HOG.

### Related works

Segmentation is an important step to extract S-HOG descriptors. Recent studies on segmentation of the glomeruli are few [1, 2]. Nevertheless, there has been some research regarding segmentation of specific organs in general biomedical images, including region growing [15], level set method [16], and active contour model [17–19]. Most of these are semi-automatic, require the users’ intervention, possess no guarantee of optimality [17–19], and are highly dependent on the initial solution provided by users as input. On the other hand, the segmentation algorithm developed in this study is ensured, theoretically, to obtain the optimal solution, producing high quality segmentation. In addition to the above-mentioned methods, more recent attempts include using deep learning [20]. Deep learning typically requires great computational and time resources, whereas the proposed algorithm can work even on a standard personal computer or a laptop.

**Fig. 1:**
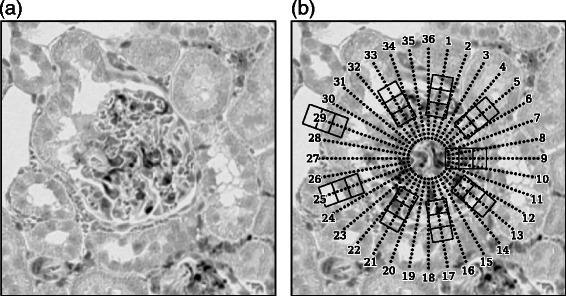
Candidate glomeruli and line segments. Segmental HOG (S-HOG) is based on the boundary of the objects of interest. If the boundary of a candidate glomerulus (Panel (**a**)) is to be located, boundary likeliness is computed at every point on *m*(=36) line segments placed uniformly in all *m* directions (Panel (**b**)). The boundary likeliness is computed at *n* points on each line segment. The *n* locations are depicted with dots in Panel (**b**)

The algorithm developed by Kvarnström et al. [21] is relevant to the proposed segmentation technique. Their algorithm for cell contour recognition is based on a dynamic program, where they first estimated the cell centers and constructed a ray from the center to each *m* direction (Fig. 1 b), where *m*=32. Then, they computed the boundary likeliness at *n* points on each ray, where they set *n*=30. Their algorithm finds a smooth contour by taking a point on each ray to connect them. To this end, they posed the following discrete optimization problem:

(1) $${} {\fontsize{8.9pt}{12.6pt}\selectfont{\begin{aligned} \max\quad\!&\sum\limits_{i=1}^{m}L_{i}(p_{i}),\qquad \text{wrt}\quad p_{1},\dots,p_{m}\in\{1,\dots,n\}, \\ \text{subject to} \quad\!& |p_{1}-p_{2}| \le\varsigma, \dots, |p_{m-1}-p_{m}|\le\varsigma, |p_{m}-p_{1}|\le\varsigma, \end{aligned}}}  $$

where $L_{i}:\{1,\dots,n\}\to {\mathbb {R}}$ denotes the boundary likeliness function obtained by the sliding window technique, and *p*_*i*_∈{1,…,*n*}(*i*=1,…,*m*) is a location on the *i*-th line segment, where the line segment is discretized into *n* points numbered with a natural number. For instance, when *p*_*i*_=1, the *i*-th vertex is at the endpoint of the *i*-th line segment closest to the center, and the vertex can move from this endpoint to the other endpoint with increasing values of *p*_*i*_. *L*_*i*_(*p*) is the boundary likeliness at the *p*-th location in the *i*-th line segment. The location *p*_*i*_ on the *i*-th line segment is more likely to be the boundary with a larger *L*_*i*_(*p*_*i*_) value.

To obtain an optimal solution, Kvarnström et al. presented two algorithms. The first algorithm poses *n* sub-problems where in each sub-problem, the initial point, and the endpoint are the same. We shall refer to this algorithm here as the *exhaustive dynamic program* (EDP). Their second algorithm is a heuristic method that is faster than the first one, but possesses no guarantee for global optimality. In this study, we developed a new segmentation algorithm, referred to as *divide & conquer dynamic program* (DCDP). Compared to Kvarnström et al’s algorithms [21], the DCDP algorithm has two advantages, as follows: not only is DCDP much faster than EDP, an exact optimal solution is always obtained; and the boundary likeliness function is trained with a machine learning technique to precisely estimate boundaries of the glomeruli.

One may consider another approach to the optimization problem (1), with a perspective that the problem is a formulation of finding a maximum a posteriori (MAP) estimation on a Markov random field (MRF) [22]. MRFs are a class of probabilistic models formulated on a graph. In the case of a problem (1), the graph has *m* nodes and forms a cycle. It is well known that the MAP estimation is efficient while using the Viterbi algorithm if the graph of the MRF is without cycles [23]. For graphs with cycles, many attempts such as LP relaxations [24] and max-product algorithms [25, 26] have been tried to compute approximate MAP estimations. Although these algorithms possess no guarantee to obtain an exact MAP estimation, they may perform well in practice. In this study, we empirically show that DCDP is much faster than these algorithms for glomeruli detection.

### Contributions

The contributions of this study are summarized as follows:

- A new descriptor called S-HOG was developed to perform a comprehensive detection of hundreds of glomeruli in images of whole kidney sections. The new descriptor is equipped with flexible blocks that can be adaptively fitted to input images to acquire robustness for detection of glomeruli.
- In our experiments, the S-HOG descriptor halved the number of false positives, a limitation of the existing state-of-the-art descriptor R-HOG, while keeping almost all true positives.
- A new segmentation technique referred to as DCDP offered high-quality segmentation outputs that were used for the construction of the blocks in S-HOG. The worst computational time of the new algorithm is equal to the fastest existing segmentation algorithm, and our experimental results reveal that the new algorithm performs overwhelmingly faster in practice.

## Methods

**Fig. 2:**
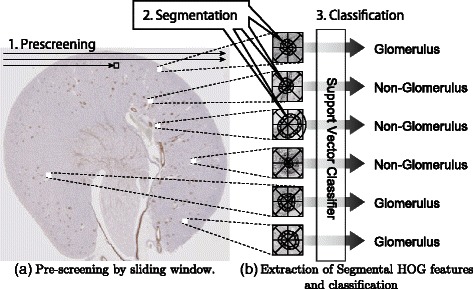
Flow of our method. In this study, a new descriptor, Segmental HOG, was developed to detect glomeruli in highly magnified microscopic images. SVM is combined with Segmental HOG to classify candidate glomeruli that passed the pre-screening stage. To do this, our detection algorithm consists of three stages. (**a**) In the pre-screening stage, candidate glomeruli are searched in the entire microscopic image. (**b**) In the segmentation stage, the boundaries of each candidate glomerulus are estimated and Segmental HOG is based on the estimated boundaries. In the classification stage, feature vectors are extracted based on the estimated segmentations, and SVM is applied to judge whether each candidate glomeruli is positive or negative
- the segmentation stage, and
- the classification stage.

In this study, a new descriptor, S-HOG, has been proposed for the detection of the glomeruli in kidney microscopic images. Segmentation of the glomeruli is needed to extract the S-HOG descriptor. For rapid detection of the glomeruli in highly enlarged microscopic images, pre-screening is performed with R-HOG, which does not require prior segmentation. The proposed method consists of the following three stages (Fig. 2):

- the pre-screening stage,

In each stage, a support vector machine (SVM) [27, 28] is used with a different type of HOG descriptor, resulting in three SVMs in total. To obtain the S-HOG descriptor, we performed segmentation of the glomeruli from the sub-images that passed the pre-screening (Fig. 2).

In what follows, we present the details of each stage, and then discuss how training datasets for each SVM are constructed and the materials used in the experiments. Finally, this section concludes with presenting a new segmentation algorithm, DCDP that is used for determining the blocks of S-HOG descriptors.

### Pre-screening

In the pre-screening stage, candidate glomeruli are detected from a kidney microscopic image using the sliding window technique. The window size is set to 200×200 in our experiments. R-HOG features, which are 512-dimensional vectors based on our selected parameter values, are extracted and judged by SVM, and non-maximal suppression is then performed to obtain the candidate glomeruli. This stage is exactly the same as in the method developed in our previous studies [10, 11]. However, the subsequent two stages dramatically reduce the false positives detected by the method. Our experiment outputs, described in the ‘Results and discussion’ section, confirm that the non-maximal suppression successfully puts the center of the window in the glomerulus, which is crucial in the segmentation step.

### Segmentation

**Fig. 3:**
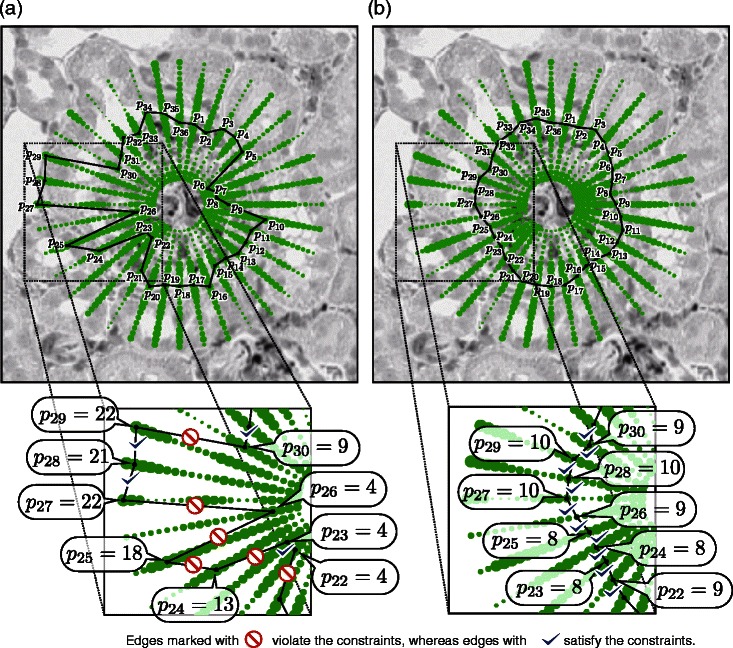
Segmentations of a candidate glomerulus. The sizes of the green dots in Panel (**a**) and Panel (**b**) represent the boundary likeliness for the candidate glomerulus shown in Fig. 2a. The value of *p*_*i*_ takes a natural number between 1 and *n*(=22) to represent the location on the *i*-th line segment. For example, in Panel (**a**), the fourth point in the 26-th line segment is selected, which is expressed as *p*_26_=4. A zigzag boundary of the glomerulus would be obtained if the points that have the largest boundary likeliness are connected naïvely without constraints, as illustrated in Panel (**a**). By considering the constraints that |*p*_*i*_−*p*_*i*+1_|≤*ς*(*i*=1,…,*n*−1) and |*p*_*m*_−*p*_1_|≤*ς* where *ς*=1, a smooth boundary can be obtained as demonstrated in Panel (**b**)

Segmentation of the glomeruli is performed on sub-images that passed the pre-screening. In the segmentation algorithm, the boundary of a glomerulus is represented by an *m*-sided polygon whose *m* vertices are restricted to lie on *m* line segments, respectively. The *m* line segments are placed uniformly, as outlined by the dotted lines in Fig. 1 b where *m*=36. To determine the location of the vertex on each line segment, the sliding window technique is employed again.^1^ The window sweeps through the line segment and computes the *boundary likeliness* at *n* locations on the line segments. In Fig. 1 b, the boundary likeliness *L*_*i*_ (*i*=1,…,*m*) is computed at every dotted location. How *L*_*i*_ is computed is discussed at the end of this ‘Segmentation’ subsection. We set the length of the line segment to 63 pixels, where the endpoint closest to the center of the image is 17 pixels away from the center. The length between adjacent dots along a line segment is equal to 3 pixels, resulting to *n*=22 dots on each line segment. In total, the boundary likeliness is computed at *m* *n*(=36×22=792) locations. The values of the boundary likeliness are depicted by the green dots in Fig. 3 a. Larger marks have higher boundary likeliness. To determine the vertices of the *m*-sided polygon, one might consider naïvely locating the points that achieve the highest boundary likeliness on each line segment. However, this approach often yields an extremely zigzag boundary (Fig. 3 a).

To obtain a smoother boundary, Kvarnström et al. [21] imposed a constraint that suppresses distant adjacent vertices, and they established the maximization problem (1). Although our implementation of the boundary likeliness is different, the formulation of the problem to find an *m*-sided polygon is the same as (1), where in our experiments, *ς* is set to *ς*=1. Fig. 3 shows an example of the solution to the optimization problem. The *m*-sided polygon shown in Fig. 3 b is the optimal solution to the maximization problem in (1). Compared to the solution without the constraint (Fig. 3 a), it is apparent that the estimated boundary is formfitting to the true boundary by introducing the constraint. The details of the new algorithm for finding the optimal solution to (1) developed in this study is presented at the end of this section.

**Computation of boundary likeliness** ***L***_***i***_ **(·)** The sliding window technique is employed in order to determine the vertices of the *m*-sided polygon described above. The window size in this stage is set to 30×15 pixels, and the windows sweep through the line segments (Fig. 1 b). Each time the sliding window moves, a feature descriptor is computed from the window and is applied with linear SVM to compute the SVM score, which is what we refer to as the boundary likeliness *L*_*i*_(·) (Fig. 3). The SVM scores of *m* vertices, *L*_*i*_(1),…,*L*_*i*_(*n*), are then obtained for *i*=1,…,*m*, and integrated in the maximization problem (1).

The HOG feature is adopted as the descriptor to compute the boundary likeliness. Each window is divided into three blocks, as shown in Fig. 1 b. This division design is from an observation that some glomeruli are surrounded with a thick Bowman’s capsule, and that the middle block is expected to capture this glomerular capsule. The statistics of nine discretely oriented gradients are computed in each block, producing a 27-dimensional feature vector.

### Classification with the S-HOG descriptor

**Fig. 4:**
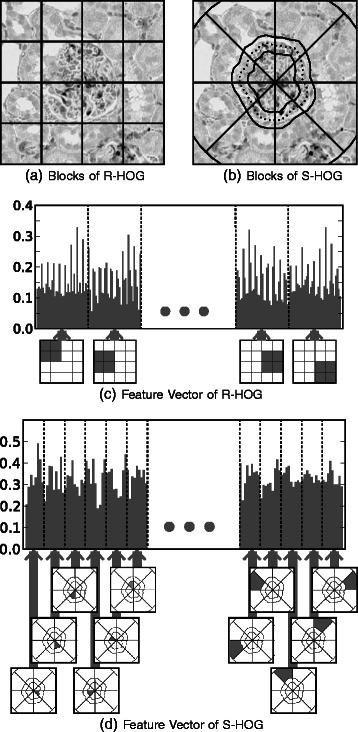
Rectangular HOG and Segmental HOG. R-HOG has been used for object detection in many applications. R-HOG is the concatenation of statistics in each block in a grid that divides a rectangular region (**a**) and (**b**). On the other hand, the blocks of the proposed descriptor, S-HOG, are based on the segmentation of the object of interest (**b**) and (**d**)

Candidate glomeruli obtained via pre-screening are classified using the proposed S-HOG descriptor. S-HOG exploits the glomerulus boundary located in the segmentation stage to generate 24 non-overlapping blocks, as shown in Fig. 4 b.

Various types of glomeruli are observed on kidney microscopic images; some of them are surrounded by a thick Bowman’s capsule. To effectively exploit this characteristic, the circle containing a candidate glomerulus is divided into the following three zones: the inner zone, middle zone, and outer zone. We divide the circle into eight disjoint sectors, and take the intersection of each zone and each sector to get 24 non-overlapping blocks (Fig. 4 b), and gradients are then histogrammed for each block (Fig. 4 d). In our experiments, we employed nine discretized oriented gradients, and SVM was applied to S-HOG feature vectors to discriminate between glomeruli and other regions.

### Construction of training data

A total of three linear SVMs are used, one each for the pre-screening, segmentation, and classification stage, respectively. A training dataset is required for each of the three SVMs. Details on the construction of each training data set are given below.

**Training data for the pre-screening stage** Each example in the training data for pre-screening is a 200×200 sub-image. A positive example contains a glomerulus in the center of the sub-image, while a negative example does not. To gather these samples, the locations of the glomeruli within the whole-kidney-section images used for training are first annotated manually. Small glomeruli whose diameters are less than 50 pixels were ignored. Positive examples are the sub-images from 200×200 bounding boxes containing an annotated glomerulus in the center. Negative examples are 200×200 sub-images picked from random locations on the kidney microscopic images. From each sample, a 512-dimensional R-HOG descriptor is extracted.

**Training data for the segmentation stage** As described in the ‘Segmentation’ subsection, the boundary likeliness is computed in every position of the *m* line segment. This boundary likeliness is the SVM score. The position lying on the true boundary of a glomerulus is considered a positive example for the SVM, and the other positions are negative examples. To construct the training data for segmentation, the positive sub-images in the training dataset for pre-screening are reused.

**Training data for the classification stage** Examples used in the training data for pre-screening are used again for training in the classification stage, but with a different set of features extracted via S-HOG. For each training data sample, the previously described segmentation algorithm estimates the boundary of the glomerulus. Based on the estimated boundary, the statistics of oriented gradients are computed to obtain S-HOG feature vectors. This procedure is done for both positive and negative examples, even though negative examples do not contain a glomerulus.

### Materials

The images used in the present study had been generated in a previous study [10], and only an overview is given in this subsection.

Twenty male 6-week-old SD and SDT rats were purchased from CLEA, Inc. (Tokyo, Japan) and were housed with a 12-h light-dark cycle and free access to water and chow.

At 16 or 24 weeks of age, SD and SDT rats (The number of rats is five for each group) were euthanized under ether anesthesia. Their kidneys were removed and immediately fixed in 10 % neutralized buffered formalin. The formalin-fixed kidneys were embedded in paraffin. For immunohistochemistry, kidney paraffin sections were deparaffinized and incubated overnight at 4 °C with anti-desmin mouse monoclonal antibody (Dako, Glostrup, Denmark) followed by horseradish peroxidase-conjugated secondary antibody (anti-mouse immunoglobulin goat polyclonal antibody; Nichirei, Tokyo, Japan). The sections were stained brown with 3,3’-diaminobenzidine. Whole slide images of the sections were obtained with Aperio Scan Scope XT (Leica Microsystems, Wetzlar, Germany). All animal experiments were performed in accordance with the Act on Welfare and Management of Animals and the institutional guidelines, and approved by the institutional Committee of Animal Experiments of New Drug Development Research Center Inc. (Hokkaido, Japan).

A total of 20 whole-kidney-section images were used in our experiments. The image sizes were 9,849×10,944 pixels in average. Each image was from one of four groups: 16-week-old SD rats, 16-week-old SDT rats, 24-week-old SD rats, and 24-week-old SDT rats. Henceforth, for simplicity, we will refer to them as 16SD, 16SDT, 24SD, 24SDT, respectively, each group containing five images. For performance evaluation, we manually annotated every glomerulus in the images. We divided the image set into five subsets: Set A, Set B, Set C, Set D, and Set E. Each subset consists of a 16SD image, a 16SDT image, a 24SD image, and a 24SDT image. For assessment of detection performance, the position of every glomerulus in the images is annotated, and for evaluation of segmentation performance, the areas of the glomeruli in Set A and Set B are located manually using a graphics software.

### Divide & conquer dynamic program (DCDP)

Herein, a new algorithm named Divide & Conquer Dynamic Program (DCDP) for solving the optimization problem (1) is presented. The new algorithm DCDP also takes *O*(*n* ^2^ *m* *ς*) computational time in worst-case scenarios, although the new algorithm solves the same problem much faster than EDP, as presented in the ‘Results and discussion’ section.

Let us denote the objective function by *J*(***p***), i.e., $J({\boldsymbol {p}}):=\sum _{i=1}^{m}L_{i}(p_{i})$, and observe that the problem (1) can be solved in *O*(*n* *m* *ς*) computational time by a dynamic program if one of the constraints |*p*_*m*_−*p*_1_|≤*ς* is disregarded. The idea to devise the new algorithm is based on the following fact: Suppose ${\boldsymbol {p}}_{0}^{\star }$ is an optimal solution that maximizes *J*(·) without the constraint |*p*_*m*_−*p*_1_|≤*ς*. Then if ${\boldsymbol {p}}_{0}^{\star }$ is a feasible solution for the original problem (1), it is also an optimal solution.

To express this idea mathematically, let us define

$${} \begin{aligned} {\mathcal{S}}({\mathcal{I}}) :=&\left\{ {\boldsymbol{p}} = \left(p_{1},\dots,p_{m}\right) \in{\mathbb{N}}_{n}^{m}\,| \right. \\& \left.p_{m}\in{\mathcal{I}}, \, |p_{1}-p_{2}|\le\varsigma, \dots, |p_{m-1}-p_{m}|\le\varsigma,\right.\\ & \left. |p_{m}-p_{1}|\le\varsigma \right\}. \end{aligned} $$

for ${\mathcal {I}}\subseteq {\mathbb {N}}_{n}$, where ${\mathbb {N}}_{n} := \left \{1,\ldots,n\right \}$. Note that ${\mathcal {S}}({\mathbb {N}}_{n})$ is the feasible region of the original problem (1). The goal of DCDP is to find an optimal solution

$${\boldsymbol{p}}^{\star} \in \operatornamewithlimits{argmax}_{{\boldsymbol{p}}\in{\mathcal{S}}({\mathbb{N}}_{n})} J({\boldsymbol{p}}). $$

Dynamic program (DP) cannot solve this problem directly owing to the existence of the constraint |*p*_*m*_−*p*_1_|≤*ς*. To use DP, we consider finding the maximizer of *J*(***p***) from a relaxed region ${\mathcal {S}}_{\mathrm {L}}({\mathbb {N}}_{n})$, where ${\mathcal {S}}_{\mathrm {L}}({\mathcal {I}})$ is defined as

$${} \begin{aligned} {\mathcal{S}}_{\mathrm{L}}({\mathcal{I}}) :=&\left\{ {\boldsymbol{p}} = \left(p_{1},\dots,p_{m}\right) \in{\mathbb{N}}_{n}^{m}\,| \right.\\ & \left.\,\, p_{m}\in{\mathcal{I}}, \, p_{1}\in{\mathcal{I}}+\{-\varsigma,\dots,+\varsigma\}, \, |p_{1}-p_{2}|\le\varsigma,\right. \\ & \left. \dots, |p_{m-1}-p_{m}|\le\varsigma \right\}\!, \end{aligned} $$

where the operator + denotes that for any two sets ${\mathcal {I}}$ and ${\mathcal {J}}$, ${\mathcal {I}}+{\mathcal {J}}:=\{i+j\,|\,i\in {\mathcal {I}},\, j\in {\mathcal {J}}\}$. Note that ${\mathcal {S}}({\mathcal {I}})\subseteq {\mathcal {S}}_{\mathrm {L}}({\mathcal {I}})$. The strategy of DCDP is to first find the solution of the relaxed problem,

$${\boldsymbol{p}}_{0}^{\star} \in \operatornamewithlimits{argmax}_{{\boldsymbol{p}}\in{\mathcal{S}}_{\mathrm{L}}({\mathbb{N}}_{n})}J({\boldsymbol{p}}) $$

and then check the feasibility: if ${\boldsymbol {p}}_{0}^{\star }\in {\mathcal {S}}({\mathbb {N}}_{n})$, then ${\boldsymbol {p}}_{0}^{\star }$ is the optimal solution of the original problem (1). If ${\boldsymbol {p}}_{0}^{\star }\not \in {\mathcal {S}}({\mathbb {N}}_{n})$, then the set ${\mathbb {N}}_{n}$ is divided into ${\mathcal {I}}_{1}$ and ${\mathcal {I}}_{2}$ (i.e. ${\mathcal {I}}_{1}\cup {\mathcal {I}}_{2} = {\mathbb {N}}_{n}$), and the following two sub-problems are solved:

$${\boldsymbol{p}}_{1}^{\star} \in\operatornamewithlimits{argmax}_{{\boldsymbol{p}}\in{\mathcal{S}}({\mathcal{I}}_{1})}J({\boldsymbol{p}}) \qquad\text{and}\qquad {\boldsymbol{p}}_{2}^{\star} \in\operatornamewithlimits{argmax}_{{\boldsymbol{p}}\in{\mathcal{S}}({\mathcal{I}}_{2})}J({\boldsymbol{p}}). $$

Notice that the original feasible region, ${\mathcal {S}}({\mathbb {N}}_{n})$, is the sum of the two regions, ${\mathcal {S}}({\mathcal {I}}_{1})$ and ${\mathcal {S}}({\mathcal {I}}_{2})$. Therefore, we can take either of the two solutions, ${\boldsymbol {p}}_{1}^{\star }$ and ${\boldsymbol {p}}_{2}^{\star }$, whichever has the larger objective value. DCDP employs a divide and conquer approach that repeatedly applies the above strategy to sub-problems. The basic approach of DCDP is summarized in Algorithm 1. Invoking the function $\text {DCDP\_Basic}({\mathbb {N}}_{n})$ yields the optimal solution for the original problem. Here, the function $({\mathcal {I}}_{1},{\mathcal {I}}_{2}) := \text {Split}({\mathcal {I}}_{0})$ divides the set ${\mathcal {I}}_{0}$ into two exclusive non-empty subsets, ${\mathcal {I}}_{1}$ and ${\mathcal {I}}_{2}$.



The first step ${\boldsymbol {p}}_{0}\in \operatornamewithlimits {argmax}_{{\boldsymbol {p}}\in {\mathcal {S}}_{\mathrm {L}}({\mathcal {I}}_{0})}J({\boldsymbol {p}})$ can be performed in *O*(*n* *m* *ς*) computational time. An instance of the dynamic program is given in Algorithm 2. Note that ${\boldsymbol {p}}_{0}\in {\mathcal {S}}({\mathcal {I}}_{0})$ is always ensured if the cardinality of ${\mathcal {I}}_{0}$ is one, because the relaxed region is reduced to the unrelaxed region (i.e. ${\mathcal {S}}(\{h\}) = {\mathcal {S}}_{\mathrm {L}}(\{h\})$). The function DCDP_Basic is invoked, at most, (2*n*−1) times. This implies that the computational time in worst cases is *O*(*n* ^2^ *m* *ς*). As will be shown in the ‘Results and discussion’ section, we empirically found that the number of invoking the function recursively is much smaller than (2*n*−1).



In the text below, the pruning steps and the resulting accelerated DCDP algorithm are detailed. Mathematical proof that the DCDP algorithm is guaranteed to obtain an optimal solution is also given. This property is favorable compared to MAP estimation methods-such as LP relaxation-that does not always achieve an optimal solution. For the implementation of $\text {Split}({\mathcal {I}}_{0})$, we considered the following three schemes: *Half Split*, *Max Split*, and *Adap Split*. These splitting schemes are described at the end of this section.

#### Pruning

Pruning can accelerate the DCDP algorithm. Consider the case where the lower boundary is *ℓ*, such that

$$\begin{array}{*{20}l} \max_{{\boldsymbol{p}}\in{\mathcal{S}}({\mathbb{N}}_{n})}J({\boldsymbol{p}}) \ge \ell, \end{array} $$

is known in advance when searching for the solution in ${\mathcal {S}}({\mathcal {I}}_{0})$. If $\ell > \max _{{\boldsymbol {p}}\in {\mathcal {S}}_{\mathrm {L}}({\mathcal {I}}_{0})}J({\boldsymbol {p}})$, then no optimal solution is in ${\mathcal {S}}({\mathcal {I}}_{0})$ because

$$\begin{array}{*{20}l} \max_{{\boldsymbol{p}}\in{\mathcal{S}}({\mathbb{N}}_{n})}J({\boldsymbol{p}}) \ge \ell > \max_{{\boldsymbol{p}}\in{\mathcal{S}}_{\mathrm{L}}({\mathcal{I}}_{0})}J({\boldsymbol{p}}) \ge \max_{{\boldsymbol{p}}\in{\mathcal{S}}({\mathcal{I}}_{0})}J({\boldsymbol{p}}). \end{array} $$

Based on this fact, the pruning step is added to obtain Algorithm 3, and we have the following lemma:

##### **Lemma1**.

For any subset ${\mathcal {I}}_{0}\subseteq {\mathbb {N}}_{n}$ and $\forall \ell \in {\mathbb {R}}\cup \{-\infty \}$, when the algorithm runs with $({\boldsymbol {p}}_{0},J_{0},\ell _{0})=\text {DCDP}({\mathcal {I}}_{0},\ell)$, the returned tuple (***p***_0_,*J*_0_,*ℓ*_0_) satisfies one of the following:

- **Case G:** If $\max _{{\boldsymbol {p}}\in {\mathcal {S}}({\mathcal {I}}_{0})}J({\boldsymbol {p}})\ge \ell $, then
  $$\begin{array}{*{20}l} {\boldsymbol{p}}_{0}\in\operatornamewithlimits{argmax}_{{\boldsymbol{p}}\in{\mathcal{S}}({\mathcal{I}}_{0})}J({\boldsymbol{p}}), \quad \text{and }\quad J_{0} = \ell_{0} = J({\boldsymbol{p}}_{0}) \ge \ell. \end{array} $$
- **Case L:** If $\max _{{\boldsymbol {p}}\in {\mathcal {S}}({\mathcal {I}}_{0})}J({\boldsymbol {p}})<\ell $, then *J*_0_<*ℓ*_0_=*ℓ*.

#### Proof of Lemma 1

We shall use the following notation: For any ${\mathcal {I}}\subseteq {\mathbb {N}}_{n}$,

$$J({\mathcal{I}}) := \max\limits_{{\boldsymbol{p}}\in{\mathcal{S}}({\mathcal{I}})}J({\boldsymbol{p}}), \quad\text{and }\quad J_{\mathrm{L}}({\mathcal{I}}) := \max\limits_{{\boldsymbol{p}}\in{\mathcal{S}}_{\mathrm{L}}({\mathcal{I}})}J({\boldsymbol{p}}). $$

The following relationships will be used in this proof:

$$J({\boldsymbol{p}}_{\mathrm{L}}) \stackrel{\smash{\mathrm{(eqA)}}}{=} J_{\mathrm{L}}({\mathcal{I}}_{0}) \stackrel{\smash{\mathrm{(ineqA)}}}{\ge} J({\mathcal{I}}_{0}) \stackrel{\smash{\mathrm{(eqB)}}}{=} \max(J({\mathcal{I}}_{1}),J({\mathcal{I}}_{2})), $$

where the labels (eqA), (ineqA), and (eqB) are used to distinguish these equalities and the inequality in later descriptions of this proof. The inequality follows from the fact that ${\mathcal {S}}({\mathcal {I}}_{0})\subseteq {\mathcal {S}}_{\mathrm {L}}({\mathcal {I}}_{0})$, while the second equality eqB follows from ${\mathcal {S}}({\mathcal {I}}_{0}) = {\mathcal {S}}({\mathcal {I}}_{1}) \cup {\mathcal {S}}({\mathcal {I}}_{2})$.

We will prove the lemma by induction. For the case where $\text {card}({\mathcal {I}}_{0})=1$, observe that ${\mathcal {S}}_{\mathrm {L}}({\mathcal {I}}_{0})={\mathcal {S}}({\mathcal {I}}_{0})$, implying that

$${\boldsymbol{p}}_{\mathrm{L}}\in\operatornamewithlimits{argmax}_{{\boldsymbol{p}}\in{\mathcal{S}}({\mathcal{I}}_{0})}J({\boldsymbol{p}}) \quad\text{and }\quad J({\mathcal{I}}_{0}) = J_{\mathrm{L}}({\mathcal{I}}_{0}) = J_{0}. $$

If $J({\mathcal {I}}_{0}) = J({\boldsymbol {p}}_{\mathrm {L}})<\ell $, then by Rule A in the algorithm, *J*_0_=−*∞* and *J*_0_<*ℓ*_0_=*ℓ*. On the other hand, if *J*(***p***_L_)≥*ℓ*, then as ${\boldsymbol {p}}_{\mathrm {L}}\in \operatornamewithlimits {argmax}_{{\boldsymbol {p}}\in {\mathcal {S}}({\mathcal {I}}_{0})}J({\boldsymbol {p}})$, we have ${\boldsymbol {p}}_{\mathrm {L}}\in {\mathcal {S}}({\mathcal {I}}_{0})$. Thus, by Rule B, we have ***p***_0_=***p***_L_ and *J*_0_=*ℓ*_0_≥*ℓ*. Therefore, the lemma is true for $\text {card}({\mathcal {I}}_{0})=1$.

Let us now assume that the lemma holds for any ${\mathcal {I}}_{0}\subseteq {\mathbb {N}}_{n}$, such that $\text {card}({\mathcal {I}}_{0}) < k$, to show that the lemma is also established for any ${\mathcal {I}}_{0}$, such that $\text {card}({\mathcal {I}}_{0})=k$. Now suppose that ${\mathcal {I}}_{0}\subseteq {\mathbb {N}}_{n}$ and $\text {card}({\mathcal {I}}_{0}) = k$. The following is an exhaustive list of all possible cases:

1. $\ell > J_{\mathrm {L}}({\mathcal {I}}_{0})$,
2. $J_{\mathrm {L}}({\mathcal {I}}_{0}) \ge \ell $ and ${\boldsymbol {p}}_{\mathrm {L}}\in {\mathcal {S}}({\mathcal {I}}_{0})$,
3. $J_{\mathrm {L}}({\mathcal {I}}_{0}) \ge J({\mathcal {I}}_{0}) = J({\mathcal {I}}_{1}) = J({\mathcal {I}}_{2}) \ge \ell $ and ${\boldsymbol {p}}_{\mathrm {L}}\not \in {\mathcal {S}}({\mathcal {I}}_{0})$,
4. $J_{\mathrm {L}}({\mathcal {I}}_{0})\ge J({\mathcal {I}}_{0}) = J({\mathcal {I}}_{1}) \ge \ell > J({\mathcal {I}}_{2})$ and ${\boldsymbol {p}}_{\mathrm {L}}\not \in {\mathcal {S}}({\mathcal {I}}_{0})$,
5. $J_{\mathrm {L}}({\mathcal {I}}_{0})\ge J({\mathcal {I}}_{0}) = J({\mathcal {I}}_{1}) > J({\mathcal {I}}_{2}) \ge \ell $ and ${\boldsymbol {p}}_{\mathrm {L}}\not \in {\mathcal {S}}({\mathcal {I}}_{0})$,
6. $J_{\mathrm {L}}({\mathcal {I}}_{0}) \ge J({\mathcal {I}}_{0}) = J({\mathcal {I}}_{2}) > J({\mathcal {I}}_{1}) \ge \ell $ and ${\boldsymbol {p}}_{\mathrm {L}}\not \in {\mathcal {S}}({\mathcal {I}}_{0})$,
7. $J_{\mathrm {L}}({\mathcal {I}}_{0}) \ge J({\mathcal {I}}_{0}) = J({\mathcal {I}}_{2}) \ge \ell > J({\mathcal {I}}_{1})$ and ${\boldsymbol {p}}_{\mathrm {L}}\not \in {\mathcal {S}}({\mathcal {I}}_{0})$,
8. $J_{\mathrm {L}}({\mathcal {I}}_{0}) \ge \ell > J({\mathcal {I}}_{0})$ and ${\boldsymbol {p}}_{\mathrm {L}}\not \in {\mathcal {S}}({\mathcal {I}}_{0})$.

We shall show that for each of the seven cases above, either Case G or Case L is true.

1. As $\ell > J_{\mathrm {L}}({\mathcal {I}}_{0}) \stackrel {\smash {\mathrm {(eqA)}}}{=} J({\boldsymbol {p}}_{\mathrm {L}}) \stackrel {\smash {\mathrm {(ineqA)}}}{\ge } J({\mathcal {I}}_{0})$, then by Rule A, we have *J*_0_=−*∞* and *J*_0_<*ℓ*_0_=*ℓ*. Thus, Case L is satisfied.
2. If $J_{\mathrm {L}}({\mathcal {I}}_{0}) \ge \ell $, then *J*_(_ ***p***_L_)≥*ℓ*. Moreover, ${\boldsymbol {p}}_{\mathrm {L}}\in {\mathcal {S}}({\mathcal {I}}_{0})$ implies ${\boldsymbol {p}}_{\mathrm {L}}\in \operatornamewithlimits {argmax}_{{\boldsymbol {p}}\in {\mathcal {S}}({\mathcal {I}}_{0})}J({\boldsymbol {p}})$ and $J({\boldsymbol {p}}_{\mathrm {L}}) = J({\mathcal {I}}_{0})$. Then by Rule B, $J_{0} = J({\boldsymbol {p}}_{\mathrm {L}}) = J({\mathcal {I}}_{0}) = \ell _{0}$ and ${\boldsymbol {p}}_{0} = {\boldsymbol {p}}_{\mathrm {L}}\in \operatornamewithlimits {argmax}_{{\boldsymbol {p}}\in {\mathcal {S}}({\mathcal {I}}_{0})}J({\boldsymbol {p}})$. Therefore, Case G holds.
3. Given $J({\boldsymbol {p}}_{\mathrm {L}}) \stackrel {\smash {\mathrm {(eqA)}}}{=} J_{\mathrm {L}}({\mathcal {I}}_{0})\ge \ell $ and ${\boldsymbol {p}}_{\mathrm {L}}\not \in {\mathcal {S}}({\mathcal {I}}_{0})$, Rule C is applied. Observe that (***p***_1_,*J*_1_,*ℓ*_1_) satisfies Case G because $J({\mathcal {I}}_{1})=\max _{{\boldsymbol {p}}\in {\mathcal {S}}({\mathcal {I}}_{1})}J({\boldsymbol {p}})\ge \ell $. Thus, $J_{1}= \ell _{1}=J({\mathcal {I}}_{1})\ge \ell $. Similarly, (***p***_2_,*J*_2_,*ℓ*_2_) also satisfies Case G, and we have $J_{2}=\ell _{2}=J({\mathcal {I}}_{2})\stackrel {\smash {\mathrm {(eqB)}}}{=}J({\mathcal {I}}_{1})= J({\mathcal {I}}_{0})$. Therefore, the returned value ***p***_0_ of $\text {DCDP}({\mathcal {I}}_{0},\ell)$ is equal to ***p***_1_ or ***p***_2_, both of which are in $\operatornamewithlimits {argmax}_{{\boldsymbol {p}}\in {\mathcal {S}}({\mathcal {I}}_{0})}J({\boldsymbol {p}})$. Furthermore, we obtain $\ell _{0}=J_{0}=J_{1}=J_{2}=J({\mathcal {I}}_{0})\ge \ell $. Hence, we have Case G.
4. As with Case (3), (***p***_1_,*J*_1_,*ℓ*_1_) satisfies Case G, and we have ${\boldsymbol {p}}_{1}\in \operatornamewithlimits {argmax}_{{\boldsymbol {p}}\in {\mathcal {S}}({\mathcal {I}}_{1})}J({\boldsymbol {p}})$ and $J_{1}=\ell _{1}= J({\mathcal {I}}_{1})\ge \ell $. Hence, it follows that $\max _{{\boldsymbol {p}}\in {\mathcal {S}}({\mathcal {I}}_{2})}J({\boldsymbol {p}}_{2})= J({\mathcal {I}}_{2}) < \ell \leq \ell _{1}$, and Case L holds for (***p***_2_,*J*_2_,*ℓ*_2_) with *J*_2_<*ℓ*_2_=*ℓ*_1_. Therefore, output ***p***_0_=***p***_1_ is in $\operatornamewithlimits {argmax}_{{\boldsymbol {p}}\in {\mathcal {S}}({\mathcal {I}}_{0})}J({\boldsymbol {p}})$ because ${\mathcal {S}}({\mathcal {I}}_{1})\subset {\mathcal {S}}({\mathcal {I}}_{0})$, and *J*_0_=*J*_1_=*ℓ*_2_=*ℓ*_0_≥*ℓ*. This gives us Case G.
5. In a similar logic as in Case (4), Case G is true for (***p***_1_,*J*_1_,*ℓ*_1_), ${\boldsymbol {p}}_{1}\in \operatornamewithlimits {argmax}_{{\boldsymbol {p}}\in {\mathcal {S}}({\mathcal {I}}_{1})}J({\boldsymbol {p}})$, and $J_{1}=\ell _{1}= J({\mathcal {I}}_{1})\ge \ell $. Given that $J({\mathcal {I}}_{2}) < J({\mathcal {I}}_{1})$, then $J({\mathcal {I}}_{2})<\ell _{1}$; hence, Case L also holds for (***p***_2_,*J*_2_,*ℓ*_2_) in this sub-case. Therefore, the same conclusion from Case (4) follows.
6. As ${\boldsymbol {p}}_{\mathrm {L}}\not \in {\mathcal {S}}({\mathcal {I}}_{0})$, Rule C is implemented. Note that $\max _{{\boldsymbol {p}}\in {\mathcal {S}}({\mathcal {I}}_{1})}J({\boldsymbol {p}})=J({\mathcal {I}}_{1})\ge \ell $, (***p***_1_,*J*_1_,*ℓ*_1_) follows Case G, and we have $J_{1}=\ell _{1}=J({\mathcal {I}}_{1})\ge \ell $. This implies that $J({\mathcal {I}}_{2})>J({\mathcal {I}}_{1})=\ell _{1}$. Therefore, Case G also holds for (***p***_2_,*J*_2_,*ℓ*_2_), and we obtain $J_{2}=\ell _{2}=J({\mathcal {I}}_{2})\!> J({\mathcal {I}}_{1})=J_{1}$. Thus, $\text {DCDP}({\mathcal {I}}_{0},\ell)$ outputs ${\boldsymbol {p}}_{0} = {\boldsymbol {p}}_{2} \in \operatornamewithlimits {argmax}_{{\boldsymbol {p}}\in {\mathcal {S}}({\mathcal {I}}_{0})}J({\boldsymbol {p}})$ and *J*_0_=*J*_2_=*ℓ*_2_=*ℓ*_0_>*ℓ*. Hence, Case G holds.
7. Similar to the previous cases, we apply Rule C. Given $\ell > J({\mathcal {I}}_{1})=\max _{{\boldsymbol {p}}\in {\mathcal {S}}({\mathcal {I}}_{1})}J({\boldsymbol {p}})$, by Case L, *J*_1_<*ℓ*_1_=*ℓ*. and so it follows that $J({\mathcal {I}}_{2})\ge \ell _{1}=\ell $. Therefore, (***p***_2_,*J*_2_,*ℓ*_2_) satisfies Case G, with $J_{2}=\ell _{2}=J({\mathcal {I}}_{2})>\ell $. Hence, the returned values of $\text {DCDP}({\mathcal {I}}_{0},\ell)$ are ${\boldsymbol {p}}_{0} = {\boldsymbol {p}}_{2} \in \operatornamewithlimits {argmax}_{{\boldsymbol {p}}\in {\mathcal {S}}({\mathcal {I}}_{0})}J({\boldsymbol {p}})$ and *J*_0_=*J*_2_=*ℓ*_2_=*ℓ*_0_>*ℓ*, and Case G is satisfied.
8. Likewise, we implement Rule C for this case. Observe that because $\max (J({\mathcal {I}}_{1}),J({\mathcal {I}}_{2})) \stackrel {\smash {\mathrm {(eqB)}}}{=} J({\mathcal {I}}_{0}) <\ell $, then both (***p***_1_,*J*_1_,*ℓ*_1_) and (***p***_2_,*J*_2_,*ℓ*_2_) satisfy Case L. Thus, we have *J*_1_<*ℓ*_1_=*ℓ* and *J*_2_<*ℓ*_1_=*ℓ*_2_. Therefore, $\text {DCDP}({\mathcal {I}}_{0},\ell)$ outputs *J*_0_= max(*J*_1_,*J*_2_)<*ℓ*_2_=*ℓ*_0_=*ℓ*_1_=*ℓ*, which corresponds to Case L.

□

Finally, from Lemma 1, we can derive the following theorem, an important theoretical result of this study:

##### **Theorem1**.

The optimal solution of the problem (1) is obtained by invoking $({\boldsymbol {p}}^{\star },J^{\star },\ell ^{\star })=\text {DCDP}({\mathbb {N}}_{n},-\infty)$.

#### Splitting schemes

For the implementation of $\text {Split}({\mathcal {I}}_{0})$, we considered three schemes: *Half Split*, *Max Split*, and *Adap Split*.

##### Half split

In this scheme, the subset of indices ${\mathcal {I}}_{0}$ is simply divided into the first half and the second half. The resulting ${\mathcal {I}}_{1}$ and ${\mathcal {I}}_{2}$ are sets of consecutive integers. For instance, this scheme divides ${\mathcal {I}}_{0} = \{ 7,8,9,10,11,12 \}$ into ${\mathcal {I}}_{1} = \{ 7,8,9 \}$ and ${\mathcal {I}}_{2} = \{ 10,11,12 \}$. To increase the lower-bound *ℓ* defined earlier, a heuristic process swaps ${\mathcal {I}}_{1}$ with ${\mathcal {I}}_{2}$ if

$$\max\limits_{h\in{\mathcal{I}}_{1}}L_{m}(h) < \max\limits_{h\in{\mathcal{I}}_{2}}L_{m}(h) $$

is employed.

##### Max split

Similar to Half Split, ${\mathcal {I}}_{1}$ and ${\mathcal {I}}_{2}$ generated by Max Split are sets of consecutive integers, although the splitting points are different. The splitting point of Half Split is the center of the interval ${\mathcal {I}}_{0}$, whereas the splitting point of Max Split is given by $h_{\star }({\mathcal {I}}_{0}) := \operatornamewithlimits {argmax}_{h\in {\mathcal {I}}_{0}}L_{m}(h)$. For example, if $h_{\star }({\mathcal {I}}_{0})=8$ for ${\mathcal {I}}_{0} = \{ 7,8,9,10,11,12 \}$, this scheme outputs ${\mathcal {I}}_{1} = \{ 7,8 \}$ and ${\mathcal {I}}_{2} = \{ 9,10,11,12 \}$. In general, the resulting divisions are given by

$${} {\fontsize{8.8pt}{9.6pt}\selectfont{\begin{aligned} {\mathcal{I}}_{1} := \left\{ h\in{\mathcal{I}}_{0}\,|\, h\le h_{\star}({\mathcal{I}}_{0}) \right\}, \quad\!\! \text{and} \quad\! {\mathcal{I}}_{2}:= \left\{ h\in{\mathcal{I}}_{0}\,|\, h> h_{\star}({\mathcal{I}}_{0}) \right\}. \end{aligned}}} $$

If $\text {card}({\mathcal {I}}_{2})=0$, the entry $h_{\star }({\mathcal {I}}_{0})$ is moved from ${\mathcal {I}}_{1}$ to ${\mathcal {I}}_{2}$. A heuristic process swaps ${\mathcal {I}}_{1}$ with ${\mathcal {I}}_{2}$ if $\text {card}({\mathcal {I}}_{1}) > \text {card}({\mathcal {I}}_{2})$ is applied.

##### Adap split

Unlike the previous two schemes, Adap Split determines the splitting point adaptively using the current solution ${\boldsymbol {p}}_{\text {L,0}}:=\operatornamewithlimits {argmax}_{{\boldsymbol {p}}\in {\mathcal {S}}_{\mathrm {L}({\mathcal {I}}_{0})}}J({\boldsymbol {p}})$. Let us denote the first entry and the last entry in the vector ***p***_L,0_ as ${p^{0}_{1}}$ and ${p^{0}_{m}}$, respectively. Using Adap Split, the mean of ${p^{0}_{1}}$ and ${p^{0}_{m}}$ is set as the splitting point. For instance, if ${p^{0}_{1}}=9$, ${p^{0}_{m}}=12$ and ${\mathcal {I}}_{0}=\{7,8,9,10,11,12\}$, then this scheme divides ${\mathcal {I}}_{0}$ into ${\mathcal {I}}_{1}=\{7,8,9,10\}$ and ${\mathcal {I}}_{2}=\{11,12 \}$. In general, the resulting divisions are given by

$$\begin{aligned} {\mathcal{I}}_{1} &:= \left\{ h\in{\mathcal{I}}_{0}\,|\, h\le \frac{{p^{0}_{1}}+{p^{0}_{m}}}{2} \right\}, \quad\text{and }\quad\\ {\mathcal{I}}_{2} &:= \left\{ h\in{\mathcal{I}}_{0}\,|\, h> \frac{{p^{0}_{1}}+{p^{0}_{m}}}{2} \right\}. \end{aligned} $$

The smallest entry in ${\mathcal {I}}_{2}$ is moved to ${\mathcal {I}}_{1}$ if $\text {card}({\mathcal {I}}_{1})=0$, and the largest entry in ${\mathcal {I}}_{1}$ is moved to ${\mathcal {I}}_{2}$ if $\text {card}({\mathcal {I}}_{2})=0$. A swapping heuristic process similar to that used in Max Split is then applied.

##### Why Adap split is better

**Fig. 5:**
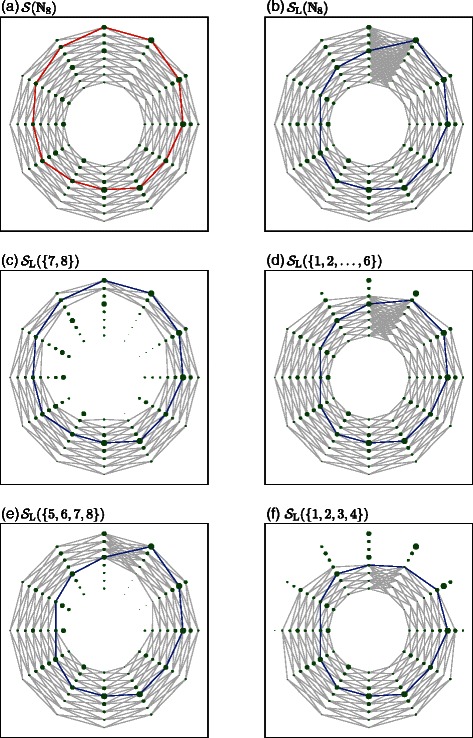
Relaxed problems in DCDP. Here, a segmentation problem (1) with (*n*,*m*,*ς*)=(8,12,1) is considered. We use an *m*-sided polygon to model the boundary of a glomerulus. The vertices are restricted to be on any of the *n* points lying on the *m* rays from the center of the glomerulus. The boundary likeliness is computed on each of the *n* points, and the configuration that maximizes the sum of the boundary likeliness is found, as described in (1). In Panel (**a**), the sizes of the green circles indicate the quantities of boundary likeliness. As in (1), the feasible configurations of the polygon are restricted to be in ${\mathcal {S}}({\mathcal {I}}_{0})$, where ${\mathcal {I}}_{0}={\mathbb {N}}_{n}$. Overlapping all feasible configurations yields the gray edges in Panel (**a**). The optimal polygon is drawn with red edges. DCDP relaxes the feasible region ${\mathcal {S}}({\mathcal {I}}_{0})$ to get ${\mathcal {S}}_{\text {L}}({\mathcal {I}}_{0})$. In Panel (**b**), the relaxed feasible region ${\mathcal {S}}_{\text {L}}({\mathcal {I}}_{0})$ is depicted. The blue polygon is the optimal configuration for the relaxed problem ${\boldsymbol {p}}_{\text {L},0}=\operatorname *{argmax}_{{\boldsymbol {p}}\in {\mathcal {S}}_{\text {L}}({\mathcal {I}}_{0})}J({\boldsymbol {p}})$. The proposed algorithm DCDP divides the problem into many sub-problems. The relaxed versions of the four sub-problems with ${\mathcal {S}}_{\text {L}}(\{7,8\})$, ${\mathcal {S}}_{\text {L}}(\{1,2,\dots,6\})$, ${\mathcal {S}}_{\text {L}}(\{5,6,7,8\})$, and ${\mathcal {S}}_{\text {L}}(\{1,2,3,4\})$ are illustrated in Panels (**c**), (**d**), (**e**), and (**f**). The blue polygons in (**c**), (**d**), (**e**), and (**f**) are the optimal solutions of the four relaxed problems, respectively. See the main text for details

Adap Split is expected to be the smartest heuristic process among the three splitting schemes. To support this claim, we illustrate the process of DCDP on a small toy problem with (*n*,*m*,*ς*)=(8,12,1), as shown in Fig. 5. The original problem and the relaxed problem are depicted in Fig. 5 a and b, respectively. When running $\text {DCDP}({\mathbb {N}}_{8},-\infty)$, where ${\mathcal {I}}_{0}={\mathbb {N}}_{8}$, it was observed that ${\boldsymbol {p}}_{\mathrm {L},0}:=\operatornamewithlimits {argmax}_{{\boldsymbol {p}}\in {\mathcal {S}}_{\mathrm {L}}({\mathcal {I}}_{0})}J({\boldsymbol {p}})\not \in {\mathcal {S}}({\mathcal {I}}_{0})$. Thereby the set ${\mathcal {I}}_{0}$ is divided into ${\mathcal {I}}_{1}$ and ${\mathcal {I}}_{2}$ to produce two new branches $\text {DCDP}({\mathcal {I}}_{1},-\infty)$ and $\text {DCDP}({\mathcal {I}}_{2},\ell _{1})$ where *ℓ*_1_ will be computed via $\text {DCDP}({\mathcal {I}}_{1},-\infty)$.

If Adap Split is employed, the two subsets are ${\mathcal {I}}_{1}=\{7,8\}$ and ${\mathcal {I}}_{2}=\{1,2,3,4,5,6\}$. In $\text {DCDP}({\mathcal {I}}_{1},-\infty)$, ${\boldsymbol {p}}_{\mathrm {L},1}:=\operatornamewithlimits {argmax}_{{\boldsymbol {p}}\in {\mathcal {S}}_{\mathrm {L}}({\mathcal {I}}_{1})}J({\boldsymbol {p}})$ is in ${\mathcal {S}} ({\mathcal {I}}_{1})$ (as shown in Fig. 5 c), implying that ***p***_L,1_ is the maximizer of *J*(***p***) over ${\mathcal {S}}({\mathcal {I}}_{1})$ and no more branching occurs. The value of *ℓ*_1_ is computed and we obtain *ℓ*_1_=*J*(***p***_L,1_)=10.1. Next, $\text {DCDP}({\mathcal {I}}_{2},\ell _{1})$ is invoked and ${\boldsymbol {p}}_{\mathrm {L},2}:=\operatornamewithlimits {argmax}_{{\boldsymbol {p}}\in {\mathcal {S}}_{\mathrm {L}}({\mathcal {I}}_{2})}J({\boldsymbol {p}}) =9.5$ is obtained (Fig. 5 d). However, *J*(***p***_L,2_)=9.5<10.1=*ℓ*_1_, which implies that the optimal solution is not in ${\mathcal {S}}({\mathcal {I}}_{2})$. Hence, ***p***_L,1_ is the optimal solution of the original problem.

On the other hand, if Half Split is applied, the set ${\mathcal {I}}_{0}={\mathbb {N}}_{8}$ is divided into ${\mathcal {I}}_{1}=\{5,6,7,8\}$ and ${\mathcal {I}}_{2}=\{1,2,3,4\}$ (Fig. 5 e and f). In $\text {DCDP}({\mathcal {I}}_{1},-\infty)$, the obtained solution of the relaxed problem is ${\boldsymbol {p}}_{\mathrm {L},1}:=\operatornamewithlimits {argmax}_{{\boldsymbol {p}}\in {\mathcal {S}}_{\mathrm {L}}({\mathcal {I}}_{1})}J({\boldsymbol {p}})={\boldsymbol {p}}_{\mathrm {L},0}$, which is, again, not in ${\mathcal {S}}({\mathcal {I}}_{1})$, leading to further branching along this sub-problem (Fig. 5 e). In fact, in our experiments discussed in the ‘Results and discussion’ section, it was observed that both Half Split and Max Split frequently encounter cases where ***p***_L,1_=***p***_L,0_ or ***p***_L,2_=***p***_L,0_. Whenever ***p***_L,1_=***p***_L,0_, additional new branches for the divisions of ${\mathcal {I}}_{1}$ are produced because ${\boldsymbol {p}}_{\mathrm {L},1}={\boldsymbol {p}}_{\mathrm {L},0}\not \in {\mathcal {S}}({\mathcal {I}}_{0})$, leading to ${\boldsymbol {p}}_{\mathrm {L},1}\not \in {\mathcal {S}}({\mathcal {I}}_{1})\subset {\mathcal {S}}({\mathcal {I}}_{0})$. Similarly, new branches for the divisions of ${\mathcal {I}}_{2}$ are also generated when ***p***_L,2_=***p***_L,0_.

In brief, the Adap Split performs better because ***p***_L,1_=***p***_L,0_ or ***p***_L,2_=***p***_L,0_ is less likely to happen in this scheme, thus resulting in less branches.

## Results and discussion

In this section, the detection performance is demonstrated by showing the experimental comparisons between S-HOG and R-HOG [10, 11].

As described in the previous section, our method has three stages: pre-screening, segmentation, and classification. Each stage uses its own SVM trained with a hyper-parameter *C*. In the classification stage, a threshold *θ* is used to classify an example; if the SVM score is over the threshold *θ*, the example is predicted as positive, otherwise, negative. For the pre-screening and classification stages, Set A was used for training SVM, and Set B was used for determining the optimal combination of (*C*,*θ*). Sets C, D, and E were used for performance evaluation. SVM for the segmentation stage provides us with the boundary likelihood function. The regularization parameter *C* for the SVM is determined via the holdout method within Set A. Seventy percent of the glomeruli in Set A are randomly selected for training, and the rest are used for validation. The resulting parameter values were (*C*,*θ*)=(10,2) for pre-screening, *C*=10 for segmentation, and (*C*,*θ*)=(10,−1.5) for classification.

### Detection performance

**Fig. 6:**
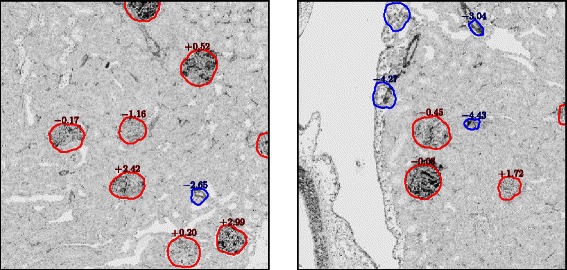
Examples of detected glomeruli. The estimated boundaries of the glomeruli are depicted with red rings. The areas surrounded with blue rings are passed through the pre-screening stage, but removed in the classification stage. The numbers are the SVM scores resulting from the classification stage. The areas with SVM scores more than *θ*=−1.5 are classified as a glomerulus. It can be observed that false positives such as vessels detected in the pre-screening stage were successfully removed in the classification stage

Figure 6 illustrates examples of detected glomeruli. In the two images, the candidate glomeruli that passed through pre-screening are depicted with rings that represent the boundaries estimated in the segmentation stage. The numbers printed above the rings are the scores produced by SVM in the classification stage. Candidate glomeruli with SVM scores below *θ*=−1.5 are excluded from the final detection results. The excluded candidates are depicted with blue rings, and the remaining glomeruli with red rings. It can be observed that non-glomerulus areas are excluded effectively and that true glomeruli are estimated correctly.

For quantitative assessment of detection performance, true positives, false positives, and false negatives have to be defined. True positive glomeruli (TPG) are identified as correctly detected glomeruli, false positive glomeruli (FPG) are wrongly detected glomeruli, and false negative glomeruli (FNG) are the ones that could not be detected. From the definitions of TPG, FPG, and FNG, we can compute for the three widely used performance measures: F-measure, precision, and recall. Precision is the ratio of TPG to the detected glomeruli (i.e. TPG/(TPG+FPG)), recall is the ratio of TPG to the true glomeruli (i.e. TPG/(TPG+FNG)), and F-measure is the harmonic mean of the Precision and Recall.

**Fig. 7:**
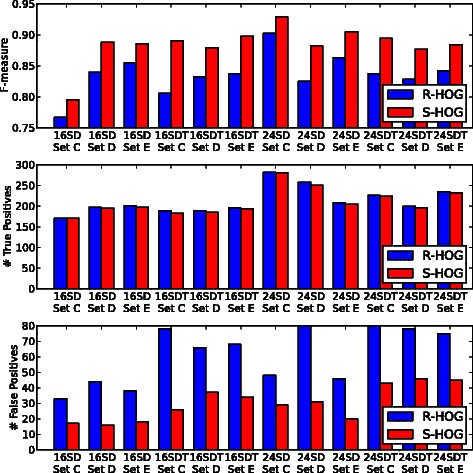
Detection performances. The proposed descriptor, S-HOG, achieves evident improvement in F-measures compared to the existing descriptor, R-HOG. With small loss of true positives, S-HOG halves false positives of R-HOG. (See subsection on ‘Detection performance’ for details)

Figure 7 shows the plots of the F-measure, Precision, and Recall for each testing image. S-HOG achieved an average of 0.866, 0.874, and 0.897 for F-measure, Precision, and Recall, respectively, whereas R-HOG obtained 0.838, 0.777, and 0.911, respectively. While applying detection methods to pathological evaluation, Precision is more important than Recall [11], and in this study, S-HOG achieved considerably higher Precision with a small sacrifice in Recall. A two-sample t-test was performed to assess the statistical differences. While no statistical difference of Recall can be detected (P-value = 3.47·10^−1^), the differences among F-measure and Precision are significant (P-values = 1.34·10^−3^ and 3.75·10^−5^, respectively).

### Segmentation performance

**Fig. 8:**
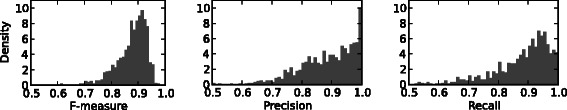
Segmentation performances. The number of glomeruli are tallied to make a histogram with the F-measure, Precision, and Recall of the pixels on the basis of comparison of true segmentation with estimated segmentation on the *x*-axes. (See ‘Segmentation performance’ subsection for details)

Herein, we discuss the performance of the segmentation algorithm. While the main purpose of the proposed method is detection, the proposed DCDP algorithm used for obtaining estimated segmentations may also be applied in some way in studies needing subsequent pathological evaluation [11]. To quantify the accuracy of the estimated areas within the predicted boundaries, 993 annotated glomeruli in Set B were used. True positive area (TPA), false positive area (FPA), and false negative area (FNA) were defined as follows: TPA is the intersection of the true area and estimated area; FPA is the relative complement of the true area in the estimated area; and FNA is the relative complement of the estimated area in the true area. For each glomerulus and its estimated area, F-measure, Precision, and Recall can be obtained by counting the pixels in the TPA, FPA, and FNA. The histograms of the F-measure, Precision, and Recall are plotted in Fig. 8, where the frequency is normalized so that the integral is one. Among the glomeruli, 90.1 *%* are estimated to have F-measures more than 0.8, ensuring reliable assessment of the medicinal effect for drug development.

**Fig. 9:**
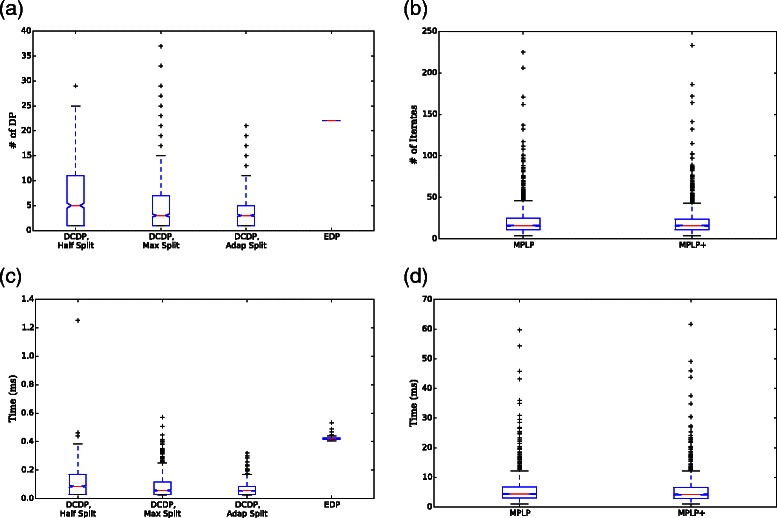
Runtime comparisons. In Panel (**a**), *n*_dp_ of DCDP with three splitting schemes and EDP is shown, where *n*_dp_ is the number of invoking the *O*(*m* *n* *ς*) DP routine. The number of iterates of MPLP and MPLP+ is plotted in Panel (**b**), where the time complexity for one iterate in MPLP and MPLP+ is *O*(*m* *n* *ς*), which is equal to one DP routine. The computational time of each algorithm is plotted in Panels (**c**) and (**d**)

The computational time of the new segmentation algorithm, DCDP, is compared with that of EDP. The two algorithms solve the same optimization problem, and both algorithms always find the same optimal solution. DCDP and EDP are implemented in C++ language, and the runtimes are measured on a Linux machine with Intel(R) Core(TM) i7 CPU and 8-GB memory. First, the number of times when the *O*(*n* *m* *ς*) DP routine was invoked, which we denote by *n*_dp_, is counted using the annotated glomeruli in Set B. Figure 9 a shows the box-plot of *n*_dp_ for all methods. While the value of *n*_dp_ for EDP is always *n*, the values for DCDP depend on the input images and the splitting schemes, Half Split, Max Split, and Adap Split. For 46.32 % of glomeruli, the optimal solutions are found within the first DP routine (i.e. *n*_dp_=1). The medians of the *n*_dp_’s when using Half Split, Max Split, and Adap Split are 5, 3, and 3, respectively. In other words, the medians of the depth of the branching tree for each scheme are 3, 2, and 2, respectively, and the respective 75th percentiles of *n*_dp_’s are 11, 7, and 5. For Adap Split, there is no case where *n*_dp_ is larger than *n*, whereas the number of glomeruli with *n*_dp_>*n* are 4 (0.40 %) and 16 (1.61 %) for Half Split and Max Split, respectively. This implies that Adap Split is the best heuristic process among the three splitting schemes.

As considered in ‘Methods’ section, Adap Split produces the same solution ***p***_L_ in the branches less frequently when compared to the other schemes. In Half Split and Max Split, the frequency (# of glomeruli) of cases where the solution ***p***_L_ in the top branch appears again in the second branches is 414 and 314, respectively. These numbers are much larger than the frequency obtained by Adap Split, which is only 97. This explains why Adap Split is faster. The actual runtime of each method is depicted in Fig. 9 c, where the medians of the computation times are 0.0866, 0.0570, 0.0560, and 0.418 msec, and the 75th percentiles of the computational times are 0.171, 0.117, 0.0856, and 0.426 msec for Half Split, Max Split, Adap Split, and EDP, respectively. As these values are proportional to the *n*_dp_’s, the ratios among the runtimes are almost the same as the ratios among the *n*_dp_’s. These results conclude that the proposed algorithm DCDP achieves an exact optimal solution much more efficiently than the existing algorithm EDP while solving the same problem, and that the Adap Scheme is the fastest splitting scheme.

The polygon model employed in this study can be reformulated as an MRF where neighboring vertices have an interaction. This perspective allows us to solve problems (1) by means of algorithms for finding a MAP estimation of MRF models. For comparison with DCDP, we examined the MPLP method [26], which is a state-of-the-art algorithm for MAP estimation of MRF. MPLP is a block coordinate-descent algorithm for minimizing the dual objective of LP relaxation. For our segmentation problem (1), the dual objective is given by

$${} {\fontsize{9.2pt}{9.6pt}\selectfont{\begin{aligned} \Psi({\boldsymbol{\lambda}}) &:= \sum\limits_{i=1}^{m}\max\limits_{p_{i}\in{\mathbb{N}}_{m}} \left(L_{i}(p_{i}) + \lambda(p_{i},i,i) + \lambda(p_{i},i-1,i) \right) \\ &- \sum\limits_{i=1}^{m}\min\limits_{(p_{i},p_{i+1})\in{\mathbb{N}}_{m}^{2}: |p_{i}-p_{i+1}|\le\varsigma} \left(\lambda(p_{i},i,i) + \lambda(p_{i+1},i,i+1) \right)\!, \end{aligned}}} $$

where ${\boldsymbol {\lambda }} := \left \{ \lambda (p_{i},i,i)\in {\mathbb {R}}\,|\,p_{i}\in {\mathbb {N}}_{n}, i\in {\mathbb {N}}_{m} \right \} \cup \left \{ \lambda (p_{i+1},\right. \left.i,i+1) \in {\mathbb {R}} \,|\,p_{i}\in {\mathbb {N}}_{n}, i\in {\mathbb {N}}_{m}\right \}$ is a set of dual variables, and we used *p*_*m*+1_ as the alias of *p*_1_ for simplicity of notation. Both *λ*(*p*_1_,0,1) and *λ*(*p*_*m*+1_,*m*,*m*+1) are aliases for *λ*(*p*_1_,*m*,1). Each variable block in the block coordinate descent is an edge. Hence, our segmentation problem (1) has *m* variable blocks, and the update rule for *i*-th edge is given by

$${} {\fontsize{8.7pt}{9.6pt}\selectfont{\begin{aligned} \lambda(p_{i},i,i) :=&-\frac{1}{2}\left(L_{i}(p_{i}) + \lambda(p_{i},i-1,i)\right) \\ &+\frac{1}{2}\max\limits_{p_{i+1}\in\left[p_{i}-\varsigma,p_{i}+\varsigma\right]} \left(L_{i+1}(p_{i+1}) + \lambda(p_{i+1},i,i+1) \right), \end{aligned}}} $$

and

$${} {\fontsize{9.1pt}{9.6pt}\selectfont{\begin{aligned} \lambda(p_{i+1},i,i+1) :=&-\frac{1}{2}\left(L_{i+1}(p_{i+1}) + \lambda(p_{i+1},i+1,i+1)\right) \\ & +\frac{1}{2}\max\limits_{p_{i}\in\left[p_{i+1}-\varsigma,p_{i+1}+\varsigma\right]} \left(L_{i}(p_{i}) + \lambda(p_{i},i,i)\right). \end{aligned}}} $$

The time complexity of one iterate is *O*(*n* *m* *ς*), which is equal to that of Algorithm 2, a dynamic program for solving each sub-problem used in DCDP. Typically, larger variable blocks reach the convergence faster in the block coordinate-descent algorithm. It can be seen easily from the update rule of *i*-th edge that the dual variables of (*m*/2) odd-numbered edges are updated simultaneously and those of (*m*/2) even-numbered edges are updated simultaneously. Then, the number of blocks is reduced to two. This algorithm is referred to as *MPLP+*. We actually implemented both MPLP and MPLP+ in C++ language and applied it to each of the 993 glomeruli images. MPLP and MPLP+ successfully obtained the optimal solutions for all the images, although MPLP and MPLP+ are not guaranteed theoretically to achieve the optimal solution. The number of iterates and the computational times are depicted in Fig. 9 b and d, respectively. Although MPLP+ has larger variable blocks than MPLP, it did not significantly improve convergence. Furthermore, it turned out that both methods are too slow to be compared with DCDP.

## Conclusions

In this study, a new descriptor, Segmental HOG, was proposed for specific glomeruli detection in microscopy images. The descriptor was based on the boundary of the glomeruli to acquire robustness for variations in intensities, sizes, and shapes. A new segmentation algorithm, DCDP, was developed to locate the boundary of possible glomeruli. Empirical results show significant improvement compared to the state-of-the-art descriptor, Rectangular HOG, for the task of glomerulus detection in microscopy images. Moreover, experimental results reveal that DCDP is much faster than the existing segmentation algorithm EDP.

Several possible uses of the proposed method can be considered. For instance, an appropriate size of the sliding window should be chosen if the proposed method is applied to microscopic images with different resolutions. In addition, while the boundary likeliness function is the same for any direction in the segmentation algorithm, different boundary likeliness functions can be used for detecting other organs depending on the orientation. For the block division of the S-HOG descriptor, 24 blocks were used in this study, as depicted in Fig. 4 c, but a different number of blocks with a different division can be used for other applications. Future studies include exploring such extensions in other applications of Segmental HOG.

## Endnote

^1^ Sliding windows are used in both pre-screening and segmentation.

## Abbreviations

R-HOG: Rectangular histogram of oriented gradients
S-HOG: Segmental histogram of oriented gradients
EDP: Exhaustive dynamic program
DCDP: Divide and conquer dynamic program
DP: Dynamic program
SVM: Support vector machine
SDT: Spontaneously diabetic torii
SD: Sprague-Dawley
16SD: 16-week-age SD rat
16SDT: 16-week-age SDT rat
24SD: 24-week-age SD rat
24SDT: 24-week-age SDT rat
TPG: True positive glomeruli
FPG: False positive glomeruli
FNG: False negative glomeruli
TNG: True negative glomeruli
TPA: True positive area
FPA: False positive area
FNA: False negative area
